# Robust Federated-Learning-Based Classifier for Smart Grid Power Quality Disturbances

**DOI:** 10.3390/s25226880

**Published:** 2025-11-11

**Authors:** Maazen Alsabaan, Abdelrhman Elsayed, Atef Bondok, Mahmoud M. Badr, Mohamed Mahmoud, Tariq Alshawi, Mohamed I. Ibrahem

**Affiliations:** 1 Department of Computer Engineering, College of Computer and Information Sciences, King Saud University, Riyadh 11451, Saudi Arabia; malsabaan@ksu.edu.sa; 2 Department of Electrical and Computer Engineering, Tennessee Tech University, Cookeville, TN 38505, USA; amelsayed42@tntech.edu; 3 Computer Science, Eastern Connecticut State University, Windham, CT 06226, USA; bondoka@easternct.edu; 4 Department of Network and Computer Security, College of Engineering, SUNY Polytechnic Institute, Utica, NY 13502, USA; badrm@sunypoly.edu; 5 Department of Electrical Engineering, Faculty of Engineering at Shoubra, Benha University, Cairo 11629, Egypt; mibrahem@augusta.edu; 6 Department of Electrical Engineering, College of Engineering, King Saud University, Riyadh 11421, Saudi Arabia; talshawi@ksu.edu.sa; 7 Department of Cyber Systems Engineering, Augusta University, Augusta, GA 30912, USA

**Keywords:** federated learning, poisoning attacks, machine learning, artificial intelligence, power quality disturbance, smart grid

## Abstract

The transition from traditional power systems to smart grids demands advanced methods for detecting and classifying Power Quality Disturbances (PQDs)—variations in voltage, current, or frequency that disrupt device performance. The rise of renewable energy and nonlinear loads, such as LED lighting, has increased PQD occurrences. While deep learning models can effectively analyze data from grid sensors to detect PQD occurrences, privacy concerns often prevent operators from sharing raw data which is necessary to train the models. To address this, Federated Learning (FL) enables collaborative model training without exposing sensitive information. However, FL’s decentralized design introduces new risks, particularly data poisoning attacks, where malicious clients corrupt model updates to degrade the global model accuracy. Despite these risks, PQD classification under FL and its vulnerability to such attacks remain largely unexplored. In this work, we develop FL-based classifiers for PQD detection and compare their performance to traditionally trained, centralized models. As expected from prior FL research, we observed a slight drop in performance: the model’s accuracy decreased from 97% (centralized) to 96% (FL), while the false alarm rate increased from 0.19% to 4%. We also emulate five poisoning scenarios, including indiscriminate attacks aimed at degrading model accuracy and class-specific attacks intended to hide particular disturbance types. Our experimental results show that the attacks are very successful in reducing the accuracy of the classifier. Furthermore, we implement a detection mechanism designed to identify and isolate corrupted client updates, preventing them from influencing the global model. Experimental results reveal that our defense substantially curtails the performance degradation induced by poisoned updates, thereby preserving the robustness of the global model against adversarial influence.

## 1. Introduction

The ongoing evolution of power infrastructure has led to the emergence of smart grids—next-generation electrical systems that integrate digital technologies, automation, and real-time data analytics to enhance the control and monitoring of electricity generation, distribution, and consumption [1,2,3]. Unlike conventional grids, smart grids incorporate components such as smart meters, sensors, communication modules, and renewable energy sources like wind and solar. These technologies enable bidirectional communication between utilities and consumers, facilitating improved fault detection, demand forecasting, and adaptive load management. The smart grid paradigm delivers multiple benefits, including greater energy efficiency, improved system reliability, faster outage response, integration of green energy sources, and active user participation in energy management, all of which contribute to a more robust and sustainable power network.

Among the primary operational challenges in smart grids are Power Quality Disturbances (PQDs)—irregularities in electrical signals—such as voltage dips, spikes, harmonics, transients, and frequency shifts that deviate from ideal power system conditions. These irregularities may arise due to load changes, hardware malfunctions, the intermittent nature of renewables, or harmonics introduced by non-linear electronic devices [4,5,6]. These disturbances can pose serious risks by damaging electronic equipment, disrupting critical operations, lowering energy efficiency, and weakening the overall reliability of the power grid, underscoring the importance of accurate and timely detection mechanisms. Several solutions for these disturbances can be implemented such as active or passive filters [7,8].

Deep learning, a subdomain of Machine learning (ML), has proven highly effective for PQD classification tasks, thanks to its capability to learn hierarchical feature representations from complex, high-resolution signal data [9,10]. Architectures such as convolutional and recurrent neural networks have shown strong performance in distinguishing between various PQD types—including sags, swells, harmonics, and interruptions—surpassing traditional signal processing techniques. However, the training of deep learning models typically requires access to large, diverse, and labeled datasets that reflect a wide range of disturbance scenarios. Acquiring such data is often challenging, as utility providers and microgrid operators may hesitate to share system-level data due to privacy concerns and fears of exposing critical infrastructure vulnerabilities.

Federated Learning (FL) provides a promising solution to this issue by enabling collaborative model training across multiple decentralized entities without sharing raw data. In FL, each participant trains a model locally and only transmits model updates or parameters to a central aggregator. This architecture allows for privacy-preserving learning while leveraging distributed datasets—a key advantage for smart grid environments where data ownership is fragmented. In the domain of smart grid, federated learning has been recently used for detection of false data [11,12], forecast future energy consumption [13,14], and intrusion detection [15]. However, FL is not without its limitations. The decentralized design introduces security challenges, particularly because participants operate independently and their datasets remain private, making them inherently untrustworthy.

One significant security risk in FL is the threat of poisoning attacks, where compromised clients deliberately corrupt local training data or labels to sabotage the global model learned by federated training. A common strategy is to mislabel a subset of a malicious client’s local data so that identical input patterns receive different labels than those used by honest clients. When the poisoned local model is aggregated with models from honest clients, the global model receives conflicting training signals (in case of classification application) and its accuracy degrades. These adversarial updates are difficult to detect, as no direct access to local data is available.

In this paper, we explore the application of FL for PQD classification and assess the vulnerability of the system to poisoning attacks. Furthermore, we propose and evaluate a defense strategy to mitigate the impact of these threats. Despite the critical role of PQD detection and the potential of FL, the combination of these two domains remains underexplored. To the best of our knowledge, this is the first study to investigate the use of FL for PQD classification and analyze the impact of poisoning attacks within this context and investigate a defense strategy.

The remainder of this paper is organized as follows. Section 2 reviews the relevant literature on PQD detection and adversarial threats in FL. Section 3 introduces the system architecture and threat models. In Section 4, we present our classifiers and evaluate their performance. Section 5 outlines the poisoning attack strategies and evaluates them, while Section 6 presents our defense strategy and evaluates it. Finally, Section 7 summarizes our findings and outlines directions for future work.

## 2. Related Work

Numerous studies have explored the use of centralized ML techniques for PQD classification, demonstrating that ML is a highly effective solution for this task. However, none of these works have examined the potential of FL in this context. Additionally, while poisoning attacks have been studied in other application domains, it has not been explored in PQD classification. In the following sections, we review the relevant literature, highlight the key limitations of existing approaches, and outline the research gaps that motivate this paper.

### 2.1. Centralized Approaches to PQD Classification Using Machine Learning

Numerous studies have explored centralized ML methods for identifying PQDs, showcasing the efficacy of deep learning in this domain. For example, in [16], Cui et. al. introduced a hybrid approach that combines the Stockwell transform for feature extraction with a convolutional neural network (CNN) for classifying multiple types of PQDs. Similarly, Ucar et. al. in [17] proposed a method that employs histogram analysis and the Discrete Wavelet Transform (DWT) for extracting features, followed by classification using an Extreme Learning Machine (ELM).

In their work [18], Topaloglu et al. introduced a CNN architecture augmented with an attention mechanism to enhance the classification accuracy of PQDs. The attention module helps the network prioritize the most informative segments of the input signal, leading to improved feature extraction and classification performance. Moreover, Yiğit et al. in [19] applied a short-time Fourier transform (STFT) to convert electrical signals into time–frequency domain representations. They then developed a hybrid model that integrates a CNN with a gated recurrent unit (GRU), where the CNN is responsible for feature extraction and the GRU component captures temporal dependencies to perform the final classification.

In [20], Wang et al. introduced an ensemble-based method for classifying PQDs, where multiple Long Short-Term Memory (LSTM) models serve as base learners. The ensemble combines their outputs using an enhanced Bagging technique. To reduce reliance on extensive expert-labeled data, they also integrated an active learning framework that selectively queries the most informative samples for annotation. In another study, Liu et al. [21] proposed a three-stage pipeline for PQD classification. Their method begins with a segmented and modified S-transform to generate detailed time–frequency representations of the signals. These representations are then fed into a CNN model to extract relevant features, which are subsequently classified using a multi-class support vector machine (SVM).

### 2.2. Adversarial Threats in Smart Grid Deep Learning Applications

Although deep learning models offer considerable advantages for smart grid applications, they are not immune to adversarial manipulation, which can lead to inaccurate predictions. In [22], Chen et al. examined the vulnerability of deep learning models used for PQD classification and load forecasting when exposed to adversarial inputs. They introduced an efficient variant of the Fast Gradient Sign Method (FGSM), originally proposed by Goodfellow et al. [23], to generate adversarial examples. Their findings revealed that the method was highly effective: over 70% of the perturbed inputs caused misclassification in a feedforward neural network (FFNN)-based PQD classifier. When used against a load forecasting system, minor manipulations to input features such as temperature settings and occupancy rates led to a 10% drop in the prediction accuracy of a recurrent neural network (RNN) model.

In another study [24], researchers explored how deep learning-based electricity fraud detection models could be evaded using adversarial examples. They developed a novel evasion attack method using a Generative Adversarial Network (GAN) to craft deceptive time-series data. This allowed high-consumption consumers to mimic the behavior of low-usage users, effectively concealing fraudulent activity. The approach proved successful across a variety of model types, including FFNNs, CNNs, and RNNs, with evasion success rates surpassing 82%, regardless of the architecture.

Additionally, Khan et al. [25] addressed the susceptibility of PQD classifiers to Trojan attacks by proposing an algorithm named Sneaky Spectral Strike ($S^{3}$). This technique embeds covert triggers into power signals, causing the model to misclassify them without being easily detected. The $S^{3}$ attack achieved over 99% success in deceiving various deep learning based PQD models. The authors also compared $S^{3}$ with three other Trojan attack strategies designed for time-series data: TrojanFlow [26], TSBA [27], and TimeTrojan-DE [28]. While the existing methods differ in trigger generation—using flow-based generative models, GANs, and evolutionary search respectively—all operate in the time domain. In contrast, $S^{3}$ employs a multi-objective heuristic search in the frequency domain, offering higher scalability and superior fooling performance with lower computational cost.

Poisoning attacks have been investigated in FL beyond the domain of PQD classification. Traditional countermeasures often rely on identifying and discarding malicious updates; however, such defenses typically assume a narrow attacker model or introduce performance degradation. For instance, ref. [29] introduced FLAME, a robust defense framework designed to eliminate attacks by adaptively injecting the minimum required noise. FLAME leverages model clustering and weight clipping to preserve model utility while successfully neutralizing malicious modifications. Evaluations on tasks like image classification, text prediction, and IoT intrusion detection confirm its effectiveness in maintaining model accuracy under attack.

Other research has focused on enhancing Byzantine resilience in distributed learning. In [30], the authors examined the robustness of Stochastic Gradient Descent (SGD) under Byzantine faults and introduced the Krum aggregation rule. Krum avoids reliance on linear combinations of client updates and is proven to maintain convergence even with faulty clients. Similarly, ref. [31] proposed Median-Krum, a hybrid of Krum and median-based aggregation, which showed strong resilience and accuracy across diverse adversarial settings. To address sybil-based poisoning threats, ref. [32] presented FoolsGold, a defense that detects malicious sybils by evaluating the similarity of client updates. It outperforms existing methods without prior knowledge of attacker distribution or auxiliary data. Lastly, the comprehensive survey in [33] offers a classification of poisoning attacks and defense techniques in FL, assessing their benefits and limitations and discussing future directions, including the intersection of privacy protection and adversarial resilience.

### 2.3. Gaps and Limitations in Existing Literature

The discussion in the preceding subsections highlights a key limitation in the current literature: no prior work has addressed the challenge of training a PQD classifier while jointly accounting for privacy and security constraints. Developing a robust and generalizable PQD classification model usually requires access to extensive and varied datasets. However, in real-world settings, this data is fragmented across different utility companies and microgrid operators. Acquiring such distributed data is difficult because these entities are typically unwilling to share raw system information, primarily due to privacy concerns and the risk of compromising grid security. Revealing operational data could unintentionally expose system vulnerabilities or patterns that adversaries might exploit to disrupt services.

To address this issue, we propose a federated learning-based framework for collaborative PQD classification. Instead of transferring raw data, each utility or microgrid operator locally trains a PQD model on its own dataset and only shares model parameters (e.g., weights or gradients) with a central server. This decentralized approach allows for the construction of a unified global model while protecting the confidentiality of local data. Moreover, this is the first work to evaluates the potential threat of poisoning attacks from malicious participants in PQD application and explores a defense.

Although the dataset used in our experiments is Identically and Independent Distributed (IID), our defense approach can also be applied in non-IID scenarios. In such cases, as demonstrated in [34], clients can be clustered into groups where data within each cluster are IID, but data across clusters are non-IID. Our proposed defense mechanism can then be effectively applied at the cluster level, ensuring robustness even under heterogeneous data distributions.

## 3. Network and Threat Models

This section begins by introducing the network model, detailing the key components of the system considered in this paper and the communication flows among them. Following that, we present the threat model, outlining the possible adversarial entities, the types of attacks they may carry out, and their intended goals.

### 3.1. Network Model

To facilitate PQD identification, electrical parameters like voltage, current, and frequency are continuously monitored and recorded at various points across the power infrastructure. Devices such as Phasor Measurement Units (PMUs), Digital Fault Recorders (DFRs), and smart meters are deployed at substations and other key grid locations to collect high-resolution, time-synchronized data. These measurements capture transient anomalies and waveform distortions, forming a critical dataset for both traditional analysis and machine learning-based classification of PQDs.

**Figure 1:**
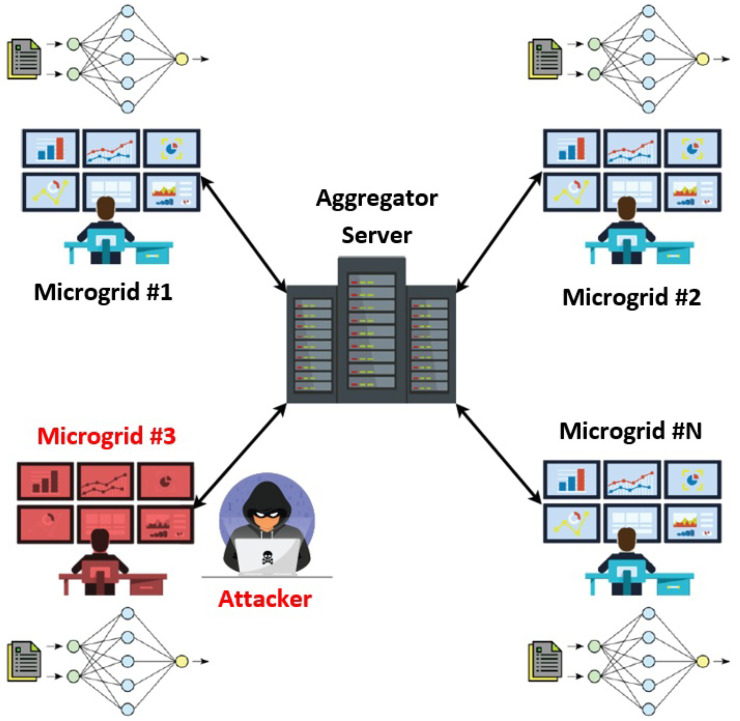
The main entities involved in our system and the communications among them.

As illustrated in Figure 1, the network model includes multiple microgrids—each representing a data owner—connected to a central server. These microgrids, typically managed by microgrid managers, are equipped with intelligent electronic devices (IEDs) and sensors that record PQD-relevant data in real time. Rather than transmitting raw data, each microgrid locally trains a machine learning model and participates in FL training, sharing only model updates with the central server. The server aggregates these updates to build a global PQD classifier, which is then redistributed to all microgrids in successive training rounds. This privacy-preserving strategy allows for accurate global model development while keeping sensitive data local. Once training is complete, microgrid managers can use the FL-based model to classify PQDs in real time based on incoming measurements. This supports rapid decision-making, such as adjusting voltage levels, activating backup systems, modifying generation sources, or balancing loads.

The central server can be viewed as the main grid control unit, which operates at a higher level and oversees coordination among multiple microgrids. Reliable communication between microgrid operators and this central server is crucial for effectively training the FL classifier. This communication may occur through various channels, including wired connections, wireless networks, or internet-based systems.

### 3.2. Threat Model

The success of the FL training process relies on stable and secure communication between the central server and the participating entities. While external threats—such as eavesdropping or data tampering during transmission—pose risks, this work assumes that such threats are mitigated through standard cryptographic techniques. Specifically, we assume the use of established encryption methods, including both symmetric and asymmetric key protocols, to protect data confidentiality and integrity during communication. Therefore, external adversaries are beyond the scope of this study. Instead, we focus on a more subtle and challenging category of threat: internal adversaries, particularly malicious microgrid managers involved in the FL process.

These participants operate independently and are not inherently trustworthy. Because FL is designed to preserve data privacy by ensuring local datasets remain on-device, it becomes difficult to verify how each client trains its local model. This privacy-preserving feature, while beneficial for data confidentiality, also creates an opportunity for malicious actors to perform stealthy attacks without detection. In particular, we consider adversaries that launch data poisoning attacks, intentionally corrupting their local training process. Such adversaries may manipulate input data, alter labels, or modify model parameters to influence the global model in a harmful way.

We investigate two common types of poisoning attacks in this context: untargeted and targeted attacks. In untargeted attacks, the adversary aims to degrade the overall performance of the global model across all disturbance categories. In contrast, targeted attacks focus on misclassifying specific classes, especially those linked to critical grid events. Successful misclassification of such events could lead to overlooked system faults or failures, resulting in serious operational or safety risks. Furthermore, adversarial participants may cooperate—sharing strategies or poisoned updates—to increase the impact of their attack. Consistent with prior studies, we assume that the number of malicious clients does not exceed 50% of the total participants, thereby ensuring that honest clients form the majority.

## 4. PQD Classifiers

In this section, we begin by describing the various machine learning model architectures employed to train the PQD classifier. Next, we outline the dataset used for training and testing, along with the performance metrics adopted to assess and compare the different architectures. We then present experimental results to evaluate the classifiers and identify the best-performing model, which will be used in the subsequent sections. Initially, we train a centralized model to serve as a baseline for comparison with the FL setting. At this stage, we assume that all FL participants behave honestly. The results obtained here will serve as a reference for the following section, where the presence of malicious participants in the FL process is considered.

### 4.1. Classifier Architectures

Building on the findings outlined in Section 2, we evaluate three distinct classifier architectures for PQD detection under centralized training. The goal is to identify the most effective model, which will then be adopted for FL experiments. The evaluated architectures include: (1) a Convolutional Neural Network (CNN), (2) a Recurrent Neural Network (RNN) incorporating Long Short-Term Memory (LSTM) units, and (3) a hybrid model that combines both CNN and RNN components. A brief overview of each architecture is provided in this section.

#### 4.1.1. CNN Model Architecture

A CNN model tailored for classifying PQDs with 100 input features and 17 output classes follows a structured, layer-based design. The input is treated as a one-dimensional sequence—suitable for a 1D CNN architecture that specializes in capturing local patterns within sequential data. The model begins with an input layer that receives a vector of 100 numeric values. This vector is reshaped to a format like ‘(100, 1)’, enabling convolutional filters to traverse the input sequence effectively.

The first processing layer is a 1D convolutional layer that applies several filters, each designed to detect specific local patterns using a sliding window approach. Filters compute dot products over short segments of the input to generate feature maps that highlight the presence of certain signal characteristics. These maps reflect how well each filter responds to different regions in the sequence.

After convolution, a non-linear activation function—typically ReLU—is applied to the feature maps, introducing the ability to learn non-linear relationships. This is followed by a pooling layer, often max pooling, which reduces the dimensionality by selecting the most prominent features within local windows. Pooling not only decreases the computational load but also improves generalization by making the model less sensitive to small variations.

Next, the resulting data structure is flattened into a single vector, which is fed into one or more fully connected (dense) layers. These layers integrate and refine the learned features, translating them into a higher-level representation. The final dense layer contains 17 neurons, corresponding to the 17 PQD categories. A softmax function is applied here to output a probability distribution over the possible classes, with the class assigned the highest probability chosen as the final prediction.

#### 4.1.2. RNN with LSTM Model Architecture

RNNs with LSTM units are well-suited for learning from sequential or time-dependent data, such as PQD signals. Unlike standard RNNs that often struggle with vanishing gradients, LSTMs are equipped with internal memory and gating mechanisms, allowing them to capture dependencies that extend over many time steps. This makes them especially useful in scenarios where input patterns unfold over time.

For a PQD classification task with 100 sequential features and 17 output categories, the model starts by accepting an input sequence shaped as ‘(100, 1)’—representing 100 time steps with a single feature each. This format feeds into an LSTM layer that processes the data step by step, maintaining both short-term and long-term context using its internal cell and hidden states. The memory structure of LSTM enables it to retain important past information while selectively forgetting irrelevant data through gates.

An initial LSTM layer with, for example, 64 units will analyze the sequence, transforming it into a set of feature representations. Optionally, additional LSTM layers can be added to build hierarchical temporal understanding. To prevent overfitting, dropout layers are often introduced after LSTM layers. These layers randomly deactivate parts of the network during training, encouraging the model to learn more generalized patterns.

After sequential processing, the temporal features are passed to one or more fully connected (dense) layers. These layers act as a classifier, translating the sequence information into a space where class decisions can be made. A typical dense layer might use ReLU activation to introduce non-linearity. More dropout can be used here as well to enhance regularization.

The final stage is a dense output layer with 17 neurons—one for each class. This layer uses the softmax function to generate a probability distribution across all classes, and the class with the highest probability becomes the model’s prediction.

#### 4.1.3. Hybrid CNN with RNN Model Architecture

A hybrid CNN-RNN model for classifying PQDs leverages the strengths of both convolutional and recurrent architectures to process sequential data more effectively. In this setup, the model begins with a 1D CNN that treats the 100 input features as a temporal sequence, applying convolutional filters to capture localized patterns and spatial dependencies such as sudden spikes, harmonics, or waveform distortions. These extracted features are then passed to an RNN layer with LSTM units, which is responsible for modeling the temporal dynamics and long-term dependencies within the signal. This structure allows the CNN to act as a powerful feature extractor, reducing noise and emphasizing relevant patterns, while the LSTM layer captures how those patterns evolve over time. The advantage of this hybrid approach lies in its ability to combine the spatial precision of CNNs with the sequential learning capacity of LSTMs, leading to a more accurate and robust PQD classifier. Ultimately, the output is fed into a dense layer with 17 softmax-activated neurons, each representing one of the target PQD classes. This architecture is especially beneficial in handling non-stationary or complex disturbance signals that exhibit both sharp local features and long-term temporal trends.

### 4.2. Centralized and FL Training

In the initial phase of our experiments, we will implement and train the three proposed PQD classification architectures using a centralized learning setup, where the entire dataset is assumed to be available to a single party. This phase serves two key purposes: (1) to evaluate and identify the most effective architecture that will be adopted for the subsequent federated learning experiments, and (2) to establish baseline performance metrics that will serve as a reference for assessing the performance of the federated models. Although federated learning introduces additional complexity, prior studies suggest that it can achieve performance levels close to those of centrally trained models. These baselines are therefore crucial for measuring the performance drop, if any, introduced by data decentralization and potential adversarial behavior.

FL is a distributed machine learning approach where multiple clients (e.g., devices or institutions) collaboratively train a shared global model without sharing their raw data. Instead, each client computes model updates (gradients or weights) locally and only these updates are sent to a central server for aggregation. Below is a mathematical explanation of the process.

Let:

- *N* be the total number of clients.
- $D_{i}$ be the local dataset on client *i* with $n_{i}=\left|D_{i}\right|$ data points.
- $n=\sum_{i=1}^{N}n_{i}$ be the total number of data samples across all clients.
- ℓ(x,y;w) be the loss function for data point (x,y) and model parameters w.

The global optimization objective is:

$$\min_{w}\;L\left(w\right)=\sum_{i=1}^{N}\frac{n_{i}}{n}\;L_{i}\left(w\right),\;\mathrm{where}\;L_{i}\left(w\right)=\frac{1}{n_{i}}\sum_{\left(x_{j},y_{j}\right)\in D_{i}}\ell \left(x_{j},y_{j};w\right).$$

At each communication round *t*, the federated averaging (FedAvg) algorithm proceeds as follows:

(1) **Server Initialization:** The global model is initialized as $w_{t}$.
(2) **Client Selection:** A subset $S_{t}\subseteq \left\{1,2,\ldots ,N\right\}$ of clients is selected.
(3) **Local Model Update:** Each selected client $i\in S_{t}$ receives the global model $w_{t}$ and updates it locally using gradient descent for *E* local epochs with batch size *B*:
  $$w_{t+1}^{i}=w_{t}-\eta \nabla L_{i}\left(w_{t}\right),$$
  where η is the local learning rate.
(4) **Model Aggregation:** The server aggregates the clients’ updates using a weighted average:
  $$w_{t+1}=\sum_{i\in S_{t}}\frac{n_{i}}{\sum_{j\in S_{t}}n_{j}}\;w_{t+1}^{i}.$$
(5) **Repeat:** Repeat the above steps for a fixed number of rounds or until convergence.

### 4.3. Evaluations

In this section, we evaluate the PQD classifier models introduced earlier, comparing two training approaches: centralized learning and federated learning. We begin by describing the PQD dataset used in our experiments, followed by an explanation of the performance metrics, and conclude with a presentation of the experimental results.

#### 4.3.1. Dataset Description

In our evaluations, we utilize the publicly available Seed Power Quality Disturbance Dataset, which is specifically designed for analyzing a wide range of power quality events. The dataset comprises signals with a fundamental frequency of 50 Hz, sampled at a rate of 5 kHz. It includes 17 distinct classes of power disturbances, with each class containing 1000 individual signals. Each signal consists of 100 samples, corresponding to a duration of 20 ms. In one approach, mathematical models defined in standards such as IEEE 1159 [35] can be used to categorize the disturbances. To ensure consistency and facilitate model convergence during training, all signal amplitudes were normalized.

The dataset is provided in two formats: (1) a MATLAB ‘.mat’ file, which contains a 3D matrix of size 1000 × 100 × 17 (signals per class × samples per signal × number of classes), and (2) 17 individual CSV files, each representing one class with a matrix of size 1000 × 100. The 17 classes include Pure Sinusoidal, Sag, Swell, Interruption, Transient, Oscillatory Transient, Harmonics, Flicker, Notch and combinations including Harmonics with Sag/Swell, Flicker with Sag/Swell, Sag with Oscillatory Transient, Swell with Oscillatory Transient, Sag with Harmonics, and Swell with Harmonics. Table 1 gives a brief description of these class types.

**Figure 2:**
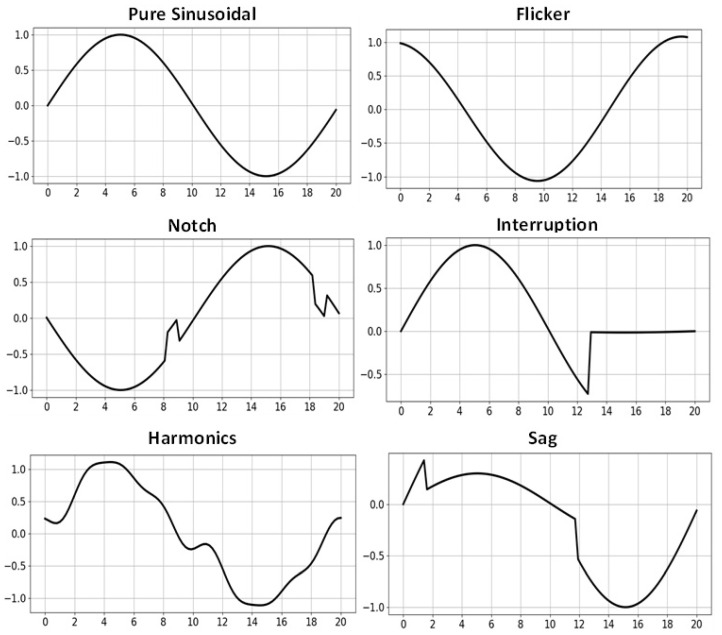
Samples of PQD data after preprocessing.

The dataset is publicly accessible via Kaggle [36,37]. Prior to model training, any samples containing missing values and outlier samples were removed to ensure data quality. For reproducibility, the dataset was shuffled using a fixed random seed and subsequently divided into training and test sets with a 80% to 20% split. Figure 2 illustrates examples of the preprocessed signals.

#### 4.3.2. Performance Metrics

In classification tasks, model performance is often evaluated using the following metrics.

*1.*  *Accuracy*

Accuracy represents the proportion of total correct predictions out of all predictions made. It can be calculated using Equation (1).

(1) $$\mathrm{Accuracy}\;\left(\mathrm{ACC}\right)=\frac{TP+TN}{TP+TN+FP+FN}$$

where:

True Positive (TP): The number of correctly predicted positive instances.
True Negative (TN): The number of correctly predicted negative instances.
False Positive (FP): The number of negative instances incorrectly classified as positive.
False Negative (FN): The number of positive instances incorrectly classified as negative.

*2.*  *False Alarm Rate (FA)*

The false alarm rate measures the proportion of negative instances that are incorrectly classified as positive.

(2) $$\mathrm{False}\;\mathrm{Alarm}\;\mathrm{Rate}\;\left(\mathrm{FA}\right)=\frac{FP}{FP+TN}$$

*3.*  *Precision*

Precision evaluates the model’s quality in positive predictions. It tells how many of the instances predicted as positive are actually positive. The higher precision indicates small false positives. Precision can be calculated using Equation (3).

(3) $$\mathrm{Precision}\;\left(\mathrm{PR}\right)=\frac{TP}{TP+FP}$$

*4.*  *F1 Score*

The F1 score is the harmonic mean of Precision and Recall, offering a balance between the two.

(4) $$F1\;\mathrm{Score}=2\cdot \frac{\mathrm{Precision}\cdot \mathrm{Recall}}{\mathrm{Precision}+\mathrm{Recall}}$$

Since Recall is defined as:

(5) $$\mathrm{Recall}=\frac{TP}{TP+FN}$$

*5.*  *Highest Difference (HD)*

The Highest Difference (HD) is the difference between the model’s accuracy and its false alarm rate. A higher HD indicates a model that not only achieves high accuracy but also maintains a low rate of false alarms, which is crucial in sensitive domains like smart grid security or intrusion detection. Generally, HD values close to 1 reflect strong performance, while lower or negative values suggest poor model behavior or misclassification issues. The HD metric is given by the following formula.

(6) HighestDifference(HD)=ACC−FA

#### 4.3.3. Experimental Results

Experiment 1: The first experiment involved training three different model architectures—CNN, RNN with LSTM units, and a hybrid CNN-RNN—using centralized learning to evaluate their effectiveness as PQD classifiers. This experiment aimed to identify the most suitable model for subsequent use in the FL setting explored in Experiment 2. The comparative results, summarized in Table 2, clearly highlight the superiority of the hybrid CNN-RNN model, which consistently delivered the best results across all six performance indicators. Notably, it achieved an accuracy of 97%, an F1-score of 96.84%, and a recall of 96.83%, while maintaining a remarkably low false alarm rate of 0.19%.

In contrast, while the standalone CNN performed reasonably well with an accuracy of 92.86%, it was outperformed by the hybrid model. The RNN-LSTM architecture, on the other hand, demonstrated the weakest performance, recording only 68.5% accuracy and the highest false alarm rate at 1.97%. In terms of model size, the standalone CNN consists of 107,791 parameters, the RNN-LSTM model includes 61,201 parameters, and the hybrid CNN-LSTM model—the most complex among them—contains a total of 145,073 parameters. These results strongly justify the adoption of the hybrid model, as it achieves higher accuracy while maintaining reasonable complexity. The hybrid architecture effectively combines the CNN’s capability to extract rich spatial features with the RNN’s proficiency in modeling temporal dependencies, resulting in a robust and reliable classifier for PQD detection.

Experiment 2: To establish a realistic and controlled experimental setup, a FL environment was carefully configured. The training dataset was evenly divided among ten clients, with each client receiving 10% of the data, simulating a decentralized training scenario. This choice is justified by the fact that each client represents a data owner, which in practice could correspond to a microgrid—hence, the number of participating clients is naturally limited. Furthermore, several existing studies, including [38,39,40], adopt a similar setup with around ten clients. Selecting ten clients also allows us to conveniently evaluate different proportions of malicious participants with 10% granularity per client, resulting in a balanced, realistic, and computationally efficient experimental configuration.

Based on the comparative analysis presented in Experiment #1, the hybrid CNN-RNN architecture was selected as the optimal model, leveraging its advanced capabilities for learning the dataset. The aggregator server first distributes an initial model with the hybrid architecture CNN-LSTM to all clients. Each client then retrains this model using its local data and sends the resulting model update back to the server, which aggregates all client updates to compute an updated global model. This process is repeated iteratively until convergence, meaning that no further improvement in model accuracy is observed. Throughout all training rounds, the clients’ models maintain the same architecture.

The system’s initial performance was assessed under ideal, attack-free conditions to serve as a reference point. The federated model achieved a high baseline accuracy of 96.05%, supported by a precision of 95% and an F1-score of 94%, reflecting strong predictive performance. Additionally, the model maintained a low false alarm rate of 4%, highlighting its robustness and reliability. These results validate the effectiveness of the selected architecture in a secure FL setting and establish a solid benchmark for evaluating the impact of adversarial behavior in the upcoming experiments.

## 5. Poisoning Attacks and Evaluations

In this section, we begin by describing the poisoning attacks used in our experiments, categorizing them into targeted and untargeted types. Untargeted attacks aim to reduce accuracy across all disturbance classes, whereas targeted attacks focus on specific classes—particularly those associated with critical grid events. Finally, we present the experimental results to assess the effectiveness of each attack strategy.

### 5.1. Description of Poisoning Attacks

This section outlines two types of poisoning attacks launched by malicious clients in a FL setting: untargeted and targeted attacks. Untargeted attacks aim to degrade the model’s overall performance by reducing accuracy across all disturbance classes. In contrast, targeted attacks specifically compromise the classifier’s accuracy on critical grid events, while maintaining normal performance on other classes. This makes targeted attacks more stealthy and dangerous, as they can evade detection and potentially lead to severe operational or safety failures in the power grid.

#### 5.1.1. Targeted Attacks

Attack #1. In this attack scenario, the adversary aims to selectively degrade the model’s accuracy for one or a few specific classes. To execute the attack effectively, the malicious clients act when the FL process is nearing convergence. At this point, the attacker trains a local model designed to misclassify the targeted class and then launches a model replacement attack [41]. This is done by crafting a local update in such a way that, once aggregated with the updates from other participants, the global model is significantly influenced—or even replaced—by the poisoned local model. Importantly, the attacker manipulates only the labels of the targeted class while preserving the labels of all other classes. In our experiment, class #8, which corresponds to the “Harmonics_with_Sag” disturbance, was selected as the attack target. All instances of this class in the attacker’s local dataset were relabeled as class 1 (“Pure_Sinusoidal”) to mislead the global model into treating this critical disturbance as a normal event.

We selected the “Harmonics_with_Sag” class because it represents a realistic and impactful disturbance that combines two critical power quality issues: voltage sag and harmonic distortion. Voltage sags cause temporary voltage dips, while harmonics—introduced by nonlinear devices —distort waveforms. Together, they can lead to transformer overheating, reduced motor efficiency, insulation damage, and malfunctioning of sensitive equipment. These effects can also disrupt protective relays and accelerate component degradation. Hence, accurately detecting this disturbance is essential to ensure system reliability and safety.

A model replacement attack in FL involves a malicious participant intentionally submitting a crafted model update designed to dominate the aggregation process and override the global model. Rather than gradually influencing the learning trajectory, the adversary manipulates the federated averaging mechanism—usually by scaling their update—to effectively force the global model to adopt their poisoned version.

The attack can be formally described by Equation (7), which defines the expected global model state $w_{*}^{t}$ at round *t*, derived from the model at the previous round $w_{*}^{t-1}$ and the collective contributions of all participating clients (*N* in total) [42], where $w_{c}^{t}$ denotes the model submitted by client *c*:

(7) $$X=w_{*}^{t}=w_{*}^{t-1}+\frac{1}{N}\sum_{c=1}^{N}\left(w_{c}^{t}-w_{*}^{t-1}\right)$$

Here, *X* represents the adversary’s intended global model. To achieve this, the malicious client *m* submits an update $w_{m}^{t}$ calculated to ensure that, after aggregation, the global model aligns with *X*. This leads to Equation (8):

The update from the malicious client *m* at time *t* is denoted as $\left(w_{c}^{t}\right)$. The resolution of Equation (7) provides Equation (8) for malicious updates:

(8) $$w_{m}^{t}=N\cdot X-N\cdot w_{*}^{t-1}-\sum_{c=1}^{N-1}\left(w_{c}^{t}-w_{*}^{t-1}\right)+w_{*}^{t-1}$$

Based on the assumptions in [43], which consider the federated system to be near convergence, the sum of deviations from honest clients becomes negligible, i.e., $\sum_{c=1}^{N-1}\left(w_{c}^{t}-w_{*}^{t-1}\right)\approx 0$. This simplifies the expression for the malicious update as follows:

(9) $$w_{m}^{t}=N\cdot X-N\cdot w_{*}^{t-1}+w_{*}^{t-1}$$

This manipulation allows the attacker to steer the global model towards a specific, potentially harmful state in a single round, especially in late stages of training when the model is close to convergence.

#### 5.1.2. Untargeted Attacks

Attack #2. In this type of poisoning attack, the adversary seeks to compromise the learning process of the global model by manipulating only the labels in their local training dataset, while leaving the input features untouched. More specifically, the attacker reassigns the labels of all local data samples to random class labels. The poisoned local model, trained on this mislabeled dataset, is then sent to the central server during the FL aggregation process. This strategy introduces inconsistencies between feature patterns and their associated labels, which can mislead the global model and hinder its ability to learn meaningful class distinctions [44]. If successful, the attack causes the FL model to misclassify various types of power quality disturbances and take improper action to the disturbance type which severely undermines the smart grid’s reliability and safety.

Attack #3. This attack shares a key similarity with Attack #2 in that the adversaries manipulate only the class labels of their local datasets, while leaving the input features unchanged. However, unlike Attack #2, Attack #3 is designed to be more deceptive and effective. In scenarios where honest clients form the majority, and their input samples closely resemble those of malicious clients—but are labeled correctly—the global model tends to learn from the honest data, reducing the adversaries’ influence. To counteract this filtering effect and increase their impact, the attackers in Attack #3 selectively choose a subset of data that is unlikely to overlap with the honest clients’ data distribution. Instead of training on the full dataset, they focus on feature subsets that are less common among honest clients, allowing them to bypass redundancy and avoid being overridden during model aggregation.

Additionally, rather than assigning labels randomly as in Attack #2, all manipulated samples are intentionally mislabeled as class #1 in Attack #3, which typically represents a “normal” or “non-disturbance” condition. This strategy serves two purposes: (1) It biases the model by overexposing it to a single class type, potentially degrading its ability to distinguish between different disturbance categories and reducing classification accuracy overall. (2) If successful, it can cause significant harm by misleading the model into misclassifying actual disturbances as normal events, preventing appropriate action from being taken. Unlike Attack #2—which may still flag a disturbance but with an incorrect type—Attack #3 deliberately masks disturbances, making it a more severe and insidious form of label poisoning.

**Figure 3:**
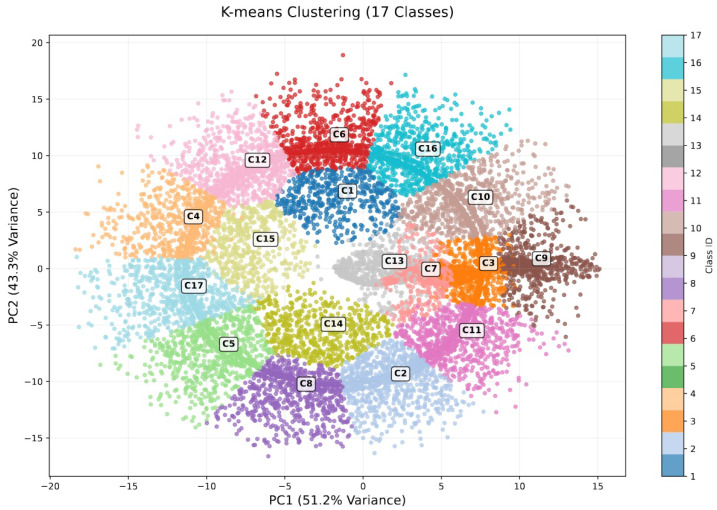
K-means clustering for the 17 classes of the dataset.

Motivated by Figure 3 that visualizes all the dataset of the 17 classes after applying K-means clustering technique, to execute Attack #3 effectively, adversarial clients first analyze their local datasets to identify data samples that are unlikely to be shared by honest clients and use them to train the local model. The goal is to select inputs that are statistically dissimilar to those held by the majority of benign participants, thereby avoiding suppression during the aggregation phase. One way to achieve this is by applying a clustering algorithm—such as K-means—to each class within the malicious client’s data. After clustering, the data samples that lie farthest from their respective cluster centroids are considered outliers and are more likely to deviate from typical data distributions seen by honest clients. These outlier samples are then used for local model training, but all are intentionally mislabeled as class #1, which represents the normal or undisturbed condition.

**Figure 4:**
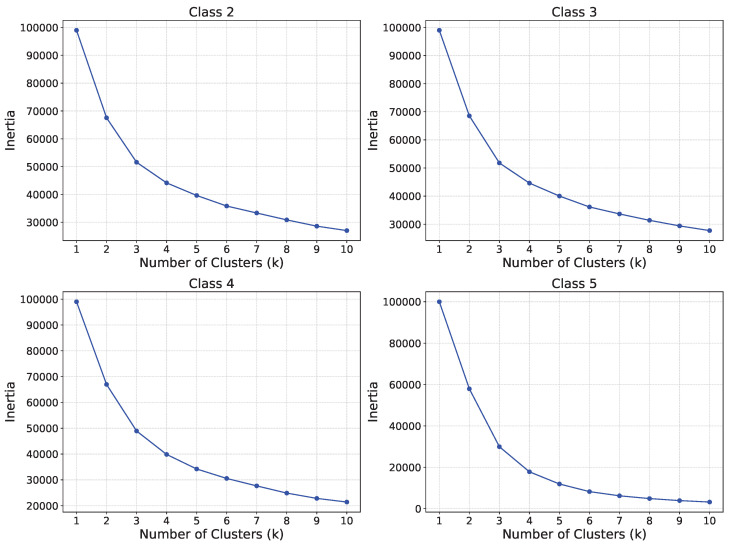
Elbow curves for classes #2, #3, #4 and #5.

**Figure 5:**
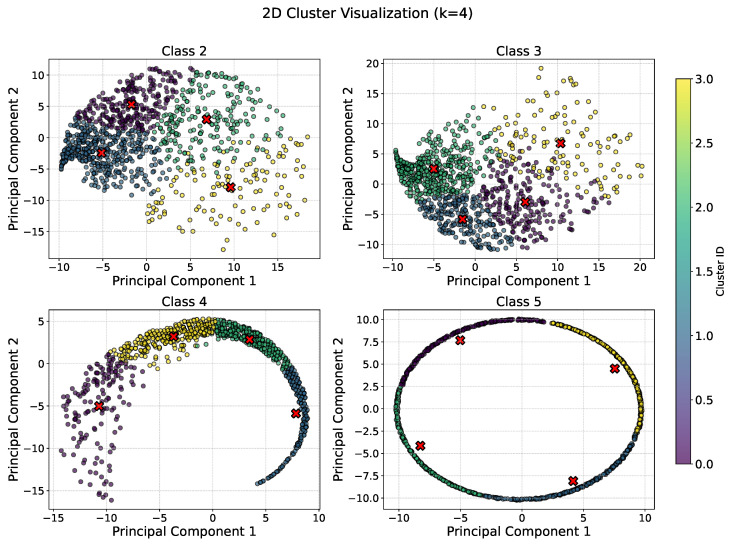
The K-means clustering plots for classes #2, #3, #4 and #5.

In our study, we apply K-means clustering to the malicious dataset and determine the optimal number of clusters (K) using the elbow method. For example, for classes labeled 2 through 5, the elbow plot indicated that K = 4 provides a reasonable balance between compactness and separation, as illustrated in Figure 4. The data distribution across clusters is visualized in Figure 5. From these clusters, we select only the samples belonging to the most distant cluster(s) in each class—those furthest from the centroid. These outlier samples are then used for local model training, but all are intentionally mislabeled as class #1, which represents the normal or undisturbed condition. These selected samples are more likely to deviate from typical data distributions seen by honest clients. After relabeling them all as class #1, they are used exclusively for training the adversarial local model, while the remaining samples are discarded. This targeted selection ensures that the manipulated data not only bypasses the influence of honest clients but also introduces a biased signal that misleads the global model into associating abnormal events with normal conditions.

Attack #4. Unlike previous attacks that manipulate only the class labels while keeping the input features unchanged, this attack adopts a different strategy by altering the feature values of the local dataset to disrupt the training process. Specifically, although the feature values in the standard training data are normalized, adversaries rescales their local features using a random scaling factor from a predefined set of values, i.e., {0.25, 0.5, −0.3, and −0.1}. This compression causes the statistical properties of the adversary’s data to differ significantly from those of the honest participants, introducing inconsistencies during the aggregation step. The poisoned model, trained on this distorted dataset, is then submitted to the federated learning process, with the aim of skewing the global model’s convergence and reducing its overall performance. By manipulating the features rather than the labels, this attack introduces a subtle yet impactful form of data poisoning that can evade traditional detection mechanisms.

Attack #5. This attack introduces a more forceful and calculated strategy compared to the earlier ones. In previous attacks, adversaries aimed to corrupt the training process by submitting models trained on tampered datasets—typically involving incorrect labels or modified features—without directly influencing the final global model. However, such approaches may be ineffective when the number of malicious clients is small, as the aggregated updates from honest clients can outweigh and suppress the impact of malicious behavior. Attack #5 addresses this limitation by integrating two techniques: it first uses Attack #3 to generate a poisoned model and then amplifies its influence through a model replacement attack. Rather than passively participating in model averaging, the adversary intentionally scales and structures their model update to override the global model during aggregation. This effectively forces the global model to adopt the attacker’s poisoned version, making the attack significantly more impactful even in the presence of a majority of honest participants. The core idea is not just to introduce noise into the system, but to decisively shift the global model’s trajectory in favor of the attacker’s objective.

### 5.2. Experimental Results

To assess how the previously described attacks influence the performance of FL and to measure their ability to degrade the accuracy of the global model, we have conducted a series of experiments using the dataset outlined in Section 4.3.1 and the evaluation metrics introduced in Section 4.3.2. This section presents and analyzes the results of those experiments. The effectiveness of each poisoning attack is quantified by the extent to which it reduces the classification accuracy of the PQD detection model, relative to the baseline performance reported in Section 4.3.3, where all participating clients are assumed to be honest. Consistent with the baseline setup, the training data was evenly divided among 10 clients, with each client receiving 10% of the total dataset. To realistically simulate varying attack intensities, the proportion of malicious clients was systematically adjusted from 10% to 40%. Furthermore, all experiments were run for 80 communication rounds to maintain a stable and comparable training environment, ensuring the observed effects are attributable to the attacks rather than experimental variability.

#### 5.2.1. Targeted Attack

**Figure 6:**
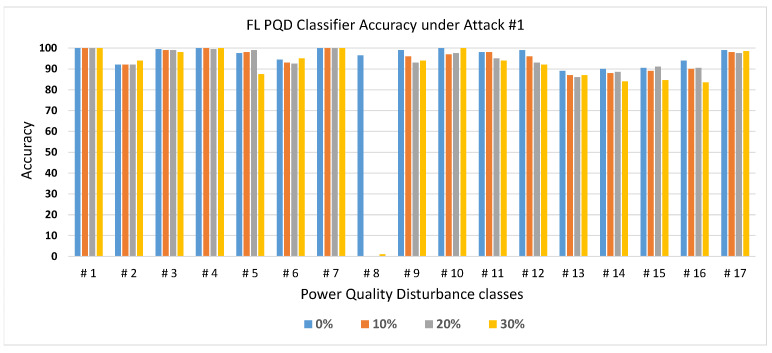
The FL PQD classifier accuracy with different percentages of malicious clients for Attack #1.

To assess the effectiveness of Attack #1, we evaluated its impact under varying proportions of malicious clients within the FL system. As previously discussed, this attack specifically targets Class #8 with the objective of significantly degrading its classification performance. To measure this, we tested the trained PQD classifier using class-specific data, allowing us to isolate and observe the model’s ability to correctly identify each individual class. The results, illustrated in Figure 6, depict the classification accuracy across all classes when 0% (baseline), 10%, 20%, and 30% of the clients are adversarial. The figure clearly demonstrates that Class #8 experiences a severe drop in accuracy—from around 96% in the absence of an attack to nearly 0% with even a small fraction of malicious participants. This drastic decline underscores the potency of the model replacement strategy, which allows adversaries to override the global model with their poisoned version by carefully scaling their local update. Consequently, even a minority of compromised clients can force the global model to converge toward a corrupted state. Meanwhile, the classification performance for non-targeted classes remains consistently high and largely unaffected, emphasizing both the precision and stealth of the attack. This selective degradation not only compromises the reliability of the PQD classifier for the targeted class but also makes detection more difficult, thereby increasing the risk to system integrity.

#### 5.2.2. Untargeted Attack

As previously discussed, untargeted attacks are designed to undermine the general effectiveness of the federated learning-based PQD classifier by reducing its overall accuracy, rather than focusing on specific classes. To assess the impact of such attacks, we have conducted a series of experiments, and the outcomes are analyzed in this section. In contrast to the evaluation approach used for targeted attacks—where classification accuracy was assessed on a per-class basis across all 17 disturbance categories—untargeted attack evaluation focuses on the global model’s overall performance. The effectiveness of an untargeted attacks is determined by the extent to which it diminishes the classifier’s overall ability to correctly identify disturbances. Greater reductions in accuracy indicate a more successful disruption.

**Figure 7:**
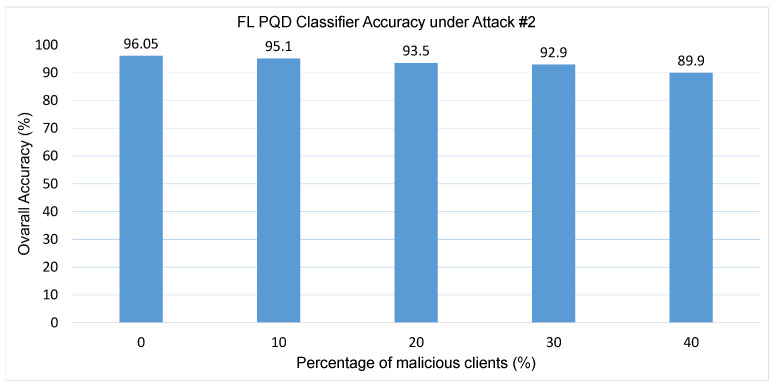
The FL PQD classifier accuracy with different percentages of malicious clients for Attack #2.

Attack #2. The impact of Attack #2 is evaluated through the results shown in Table 3 and Figure 7. Table 3 presents the performance of the PQD classifier as the number of malicious clients increases, while Figure 7 illustrates how the classifier’s overall accuracy is affected by different proportions of adversarial participants. As shown in the table, the HD metric exhibits a steady decline, decreasing from 92% to 80.6% as the attack becomes more intense. Despite this degradation, the classifier’s precision remains consistently strong—exceeding 90% even when four clients (40%) are malicious. Consequently, the F1-score, which captures the trade-off between precision and recall, remains robust throughout the experiment, only dropping slightly from 94% to 89.8%. This performance, however, is accompanied by a moderate increase in the False Alarm rate, which grows from 4% to 9.3%. Additionally, Figure 7 reveals a clear downward trend in accuracy as the percentage of malicious clients increases—from an initial 96.05% in the attack-free scenario to 89.9% when 40% of the clients are compromised. Overall, the findings indicate that although this untargeted attack introduces some level of disruption, its overall effect on the global model remains relatively limited. This can be attributed to the dominant influence of honest clients, whose accurate updates tend to dilute or neutralize the adversarial contributions during model aggregation.

**Figure 8:**
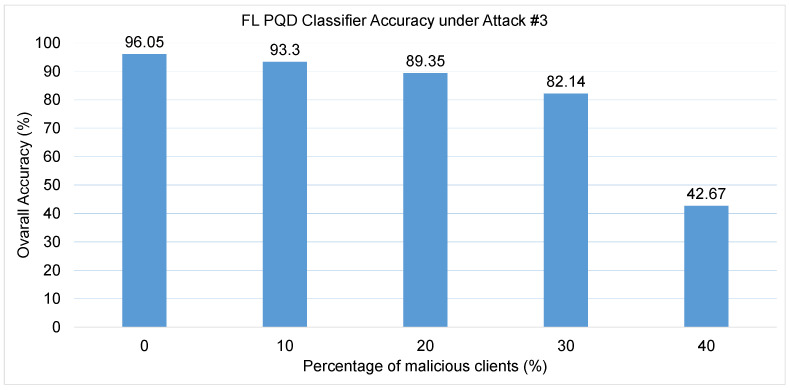
The FL PQD classifier accuracy with different percentages of malicious clients for Attack #3.

Attack #3. The effectiveness of Attack #3 is assessed using the results presented in Table 4 and Figure 8. The table outlines the PQD classifier’s performance as the proportion of malicious clients increases, while the figure tracks the corresponding changes in overall model accuracy. As shown in the table, this attack causes a sharp and consistent degradation in classifier’s performance metrics. The HD drops significantly—from a baseline of 92% under honest conditions to only 21.5% when 40% of the participants are compromised. Similarly, the F1-score, which captures the balance between precision and recall, deteriorates from 94% to just 43%, reflecting a substantial loss in predictive performance. Precision also experiences a steep decline, decreasing from 95% to 54% as the attack intensifies. Conversely, the False Alarm rate rises sharply, increasing more than fivefold—from 4% to 21%—indicating growing instability and unreliability in the classifier’s decisions.

Figure 8 further highlights the attack’s disruptive influence on classifier accuracy. In the absence of malicious clients, the classifier maintains strong performance with an accuracy of 95.7%. As adversarial participation rises to 10%, 20%, and 30%, accuracy gradually falls to 93.3%, 85.86%, and 82.14%, respectively. However, when 40% of the clients are malicious, the accuracy collapses to just 42.67%. These findings clearly demonstrate that while the PQD classifier can tolerate a limited number of adversaries with moderate performance degradation, its robustness breaks down when a large fraction of the network is compromised. Under such conditions, the classifier’s reliability is severely undermined, making the system highly vulnerable to coordinated attacks. Compared to Attack #2, this attack demonstrates significantly greater effectiveness, primarily due to its strategic approach of trained the local detector on data samples that are distinctly different from those of honest clients. Also, by assigning class one label to all data, the poisoned model introduces more confusion during training, making it harder for the global model to generalize correctly and leading to a greater degradation in classification performance.

**Figure 9:**
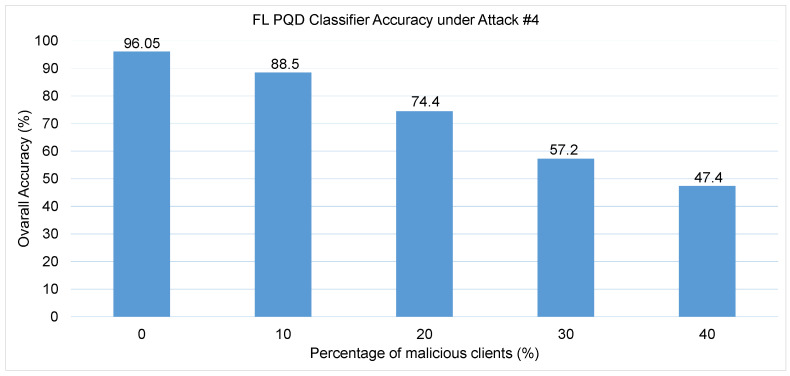
The FL PQD classifier accuracy with different percentages of malicious client for Attack #4.

Attack #4. The impact of Attack #4 is evaluated through the results summarized in Table 5 and illustrated in Figure 9. The findings reveal a pronounced decline in the PQD classifier’s performance as the proportion of malicious clients increases from 0% to 40%. Specifically, the HD metric, which initially stands at 92% in a trusted training environment, plummets to just 2.4% when 40% of the clients are adversarial. Similarly, Precision and F1-score, which begin at 95% and 94% respectively, deteriorate to 49% and 41%, reflecting a severe loss in classification reliability. Meanwhile, the FA rate surges dramatically from a modest 4% to 45%, indicating a significant spike in incorrect predictions. This pattern is mirrored in Figure 9, where the PQD classifier’s overall accuracy steadily declines as the percentage of malicious clients increases: from 88.5% at 10% attackers to 74.4% at 20%,and further down to just 57.2% at 30%, and finally 47.4% at 40%.

These results clearly demonstrate that Attack #4 can substantially degrade model performance even when a relatively small fraction of clients are compromised. Furthermore, when compared to Attacks #2 and #3, which manipulate data labels, Attack #4 proves to be more damaging—suggesting that tampering with input features introduces deeper disruption into the learning process than label flipping alone.

**Figure 10:**
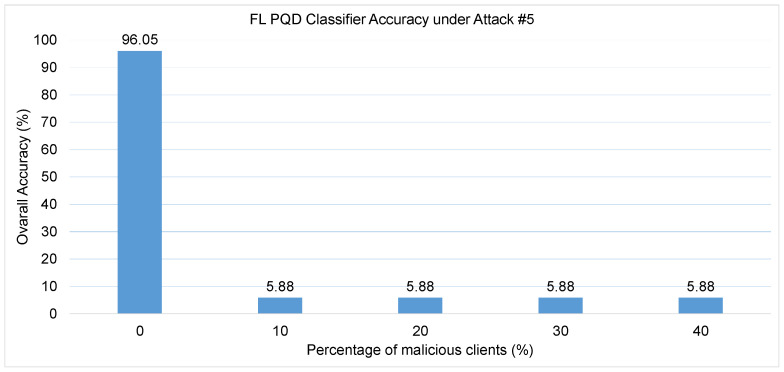
The FL PQD classifier accuracy with different percentages of malicious clients for Attack #5.

Attack #5. The effectiveness of Attack #5 is assessed using the results presented in Table 6 and Figure 10. Compared to the baseline metrics—HD of 92.05%, Precision of 95%, F1-score of 94%, and a False Alarm rate of 4%—the results indicate a complete collapse in model performance with the introduction of only a single malicious client. Both the HD and Precision sharply decline to nearly 0%, and the F1-score follows suit, dropping to approximately 1%. This dramatic impact is further confirmed by Figure 10, which shows that the classifier’s accuracy plunges from 96% to just 5.88% when only one adversarial client is involved.

**Figure 11:**
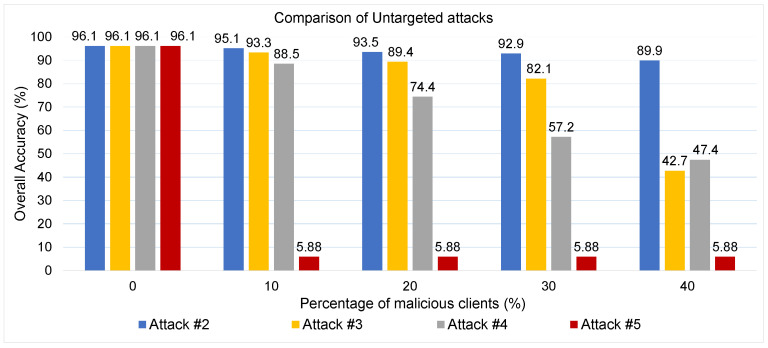
Comparison between different untargted attacks.

To further contextualize the severity of this attack, Figure 11 compares the effects of various untargeted poisoning strategies. Unlike prior attacks, where the classifier’s performance degrades gradually with an increasing number of attackers, Attack #5 exhibits an immediate and severe breakdown even with minimal adversarial participation. This is because the model replacement strategy allows a single attacker to override the global model with a fully poisoned version, bypassing the need for multiple malicious updates. In contrast, previous attacks attempted to gradually steer the model off course by injecting corrupted updates that had to contend with the influence of honest clients—making their impact more proportional to the number of attackers. When comparing the overall severity and effectiveness of untargeted attacks, Attack #5 stands out as the most destructive, as it allows an adversary to completely compromise the integrity of the global model with minimal effort by directly replacing it with a maliciously crafted version.

## 6. Defense and Evaluations

In this section, we implement a defense mechanism designed to detect and exclude poisoned updates before aggregation, preventing them from influencing the global model. The mechanism is based on the observation that a poisoned model, being trained on manipulated data, typically produces update directions (gradients or parameter changes) that differ significantly from those of honest clients. By analyzing the pairwise distances between submitted model updates, the defense algorithm can identify outliers that are likely to be malicious. If an attacker attempts to conceal their behavior by making their updates resemble those of honest clients, the effectiveness of the attack diminishes—since the closer a malicious update is to honest ones, the less influence it has in steering the global model toward the attacker’s intended target. Then, we will present experimental evaluations to assess the effectiveness of the defense strategy.

### 6.1. Defense Strategy

Our defense strategy is designed around a robust filtering mechanism that detects and excludes poisoned updates submitted by malicious clients before they can corrupt the global model. The central idea is to refine the aggregation process by employing a median-based rule, which selects the median value of each parameter across all clients’ gradient vectors. This approach inherently reduces the influence of outliers, making it particularly effective against adversarial manipulations. This defense technique is implemented by the central server of the federated learning.

Prior research in domains other than PQD classification [34,45,46,47] has shown that when client data is independently and identically distributed (IID), the gradients computed by benign clients tend to be highly consistent. While minor differences in magnitude may exist, the gradients overwhelmingly align in direction with high probability. This consistency reflects the shared optimization objective of benign participants. By contrast, malicious clients intentionally craft poisoned updates that diverge substantially from this consensus, aiming to shift the global model toward a parameter space that serves the attacker’s goal.

This sharp distinction between the directional coherence of benign updates and the divergence of adversarial ones provides a natural basis for filtering. By applying the median rule, poisoned updates are effectively suppressed without the need for explicit anomaly detection, thereby reinforcing the resilience of the federated learning process. Importantly, while this approach has been validated in other machine learning applications, its effectiveness has not yet been thoroughly tested in the context of PQD classification, where data distributions and attack surfaces may exhibit unique characteristics. Moreover, even if the dataset is not perfect and it contains some incorrectly labeled samples, our defense remains effective. This is because attackers deliberately mislabel all of their data samples, causing their model parameters to deviate significantly from those of honest clients.

The median aggregation approach operates by computing the median for each coordinate of the parameter vector. Consider a model update represented as a parameter vector G of dimension *d*. For the *i*-th parameter, the aggregated value $G_{ei}$ is obtained by taking the median (Med) of the corresponding values $G_{ni}$ submitted by the *N* participating clients, as expressed in the following equation:

(10) $$G_{ei}=\mathrm{Med}\;G_{ni},\;n\in \left[N\right]\;\mathrm{and}\;i\in \left[d\right]$$

Here, *n* indexes the clients and *i* indexes the model dimensions. The calculation of the median depends on the total number of clients *N*. When *N* is odd, the median is simply the central value in the sorted sequence of parameter values. When *N* is even, it is computed as the average of the two central values.

The defensive properties of median aggregation make it particularly effective in adversarial federated learning scenarios. The median automatically filters out extreme gradient values that deviate significantly from the honest majority, regardless of their magnitude or direction. Each parameter coordinate is processed independently, which means that malicious clients cannot strategically manipulate multiple dimensions simultaneously to bypass the defense. For the learning process, the median aggregation ensures convergence because the median prevents any single malicious client from drastically altering the gradient direction, thereby maintaining steady progress toward good model parameters throughout the training process.

### 6.2. Experimental Results

To evaluate the impact of the proposed defense on federated learning accuracy and overall performance, we have conducted experiments using the dataset described in Section 4.3.1 and assessed the results with the metrics defined in Section 4.3.2. The following analysis highlights the effectiveness of the defense in mitigating the poisoning attacks detailed in Section 5.

#### 6.2.1. Evaluations for Targeted Attack

**Figure 12:**
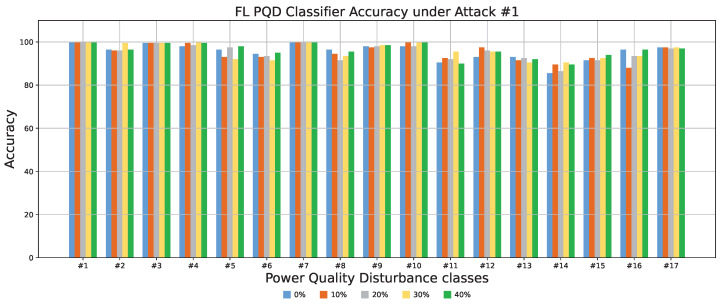
The FL PQD classifier accuracy under Attack #1 using the Median Defense.

Attack #1. Figure 12 shows the classification accuracy of the FL-based PQD model when protected by our defense. Compared with Figure 6, which depicts performance without any defense, the proposed method demonstrates a substantial improvement in robustness. Notably, the accuracy for class #8 increases from nearly 0% in the undefended case to about 93% when the proportion of malicious clients is between 10% and 40%. This gain is a direct result of the median aggregation mechanism used by our defense, which effectively emphasizes benign gradients while suppressing adversarial updates during global model training.

#### 6.2.2. Evaluations for Untargeted Attacks

**Figure 13:**
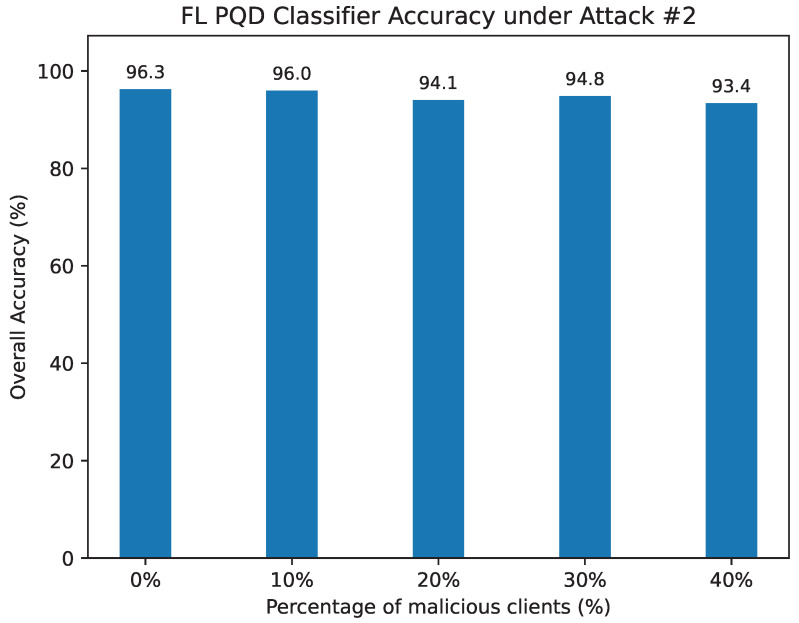
The FL PQD classifier accuracy under Attack #2 using the Median Defense.

Attack #2. The effectiveness of the proposed defense against Attack #2 is summarized in Table 7 and illustrated in Figure 13. Under our defense, the HD metric remains consistently high, decreasing only slightly from 92.26% in the benign case to 89.08% when 40% of clients are compromised. By comparison, in the absence of defense (Table 3), HD drops more noticeably, from 92% to 80.6% as adversarial participation grows. A similar stabilizing effect is observed for precision, F1-score, and false alarm rate, where the defense alleviates the degradation typically caused by poisoned updates.

For overall classification accuracy, our defense maintains strong performance, showing only a modest decline from 96.3% without attacks to 93.4% with 40% malicious clients. In contrast, the unprotected model experiences a sharper decline, falling from 96.05% to 89.9%. These findings confirm that the proposed defense substantially mitigates the adverse effects of untargeted poisoning attacks and preserves the reliability of the global model.

**Figure 14:**
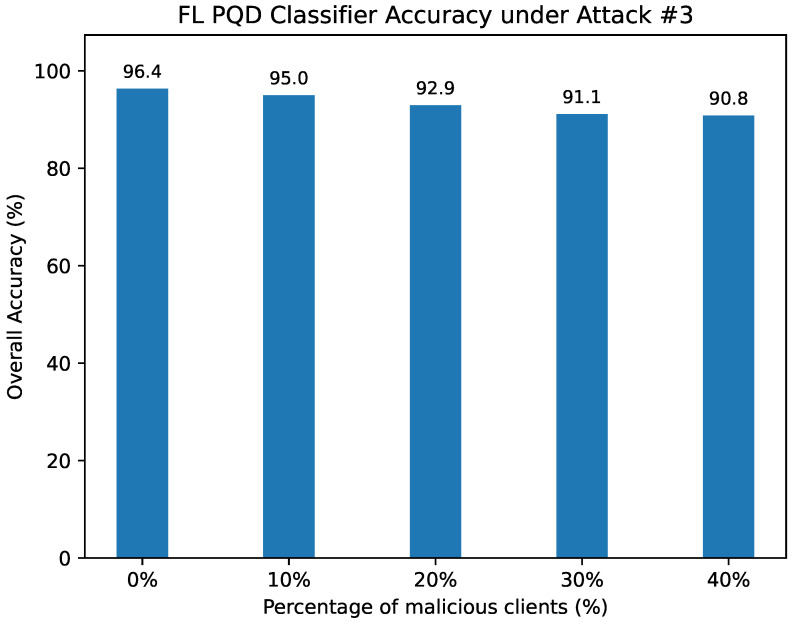
The FL PQD classifier accuracy under Attack #3 using the Median Defense.

Attack #3. Table 8 and Figure 14 report the performance of our defense under Attack #3. With the defense in place, the HD metric remains strong, showing only a modest decline from 92.26% in the benign case to 89.08% when 40% of clients are adversarial. In comparison, the unprotected model (Table 4) suffers a severe reduction, with HD falling from 92% to only 21.5% as the proportion of malicious clients grows. Precision, F1-score, and false alarm rate exhibit the same stabilizing trend, where the defense substantially mitigates the degradation caused by poisoned updates.

In terms of overall classification accuracy, our defense preserves high performance, decreasing slightly from 96.36% with no adversaries to 90.85% at 40% adversarial clients. By contrast, the absence of defense results in a dramatic accuracy drop from 95.7% to 42.67% under identical conditions. These results highlight the resilience of the median aggregation approach in countering untargeted poisoning attacks and maintaining the integrity of the global model.

**Figure 15:**
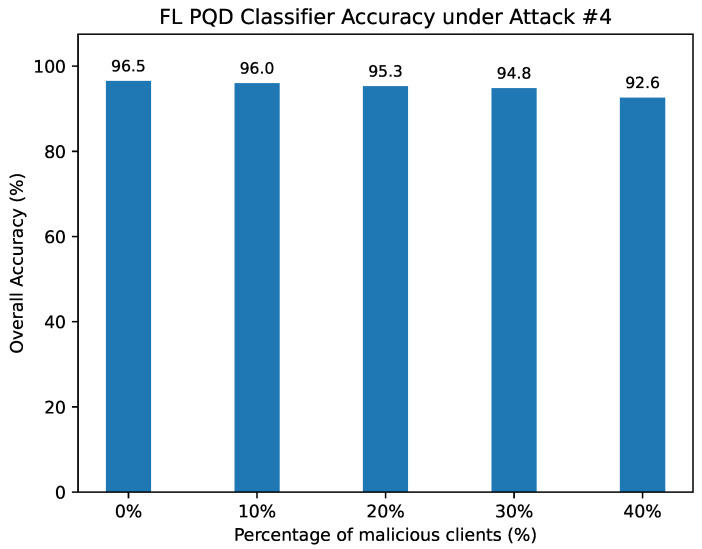
The FL PQD classifier accuracy under Attack #4 using the Median Defense.

Attack #4. Table 9 and Figure 15 present the performance of the FL-based PQD classifier under Attack #4 when protected by our defense. With this defense, the HD metric remains stable, dropping only slightly from 92.49% in the benign scenario to 88% when 40% of clients act maliciously. In sharp contrast, the model without defense (Table 5) experiences a severe collapse, with HD plunging from 92% to just 2.4% as adversarial participation increases.

Comparable trends are evident in other evaluation metrics, such as precision, F1-score, and false alarm rate. In each case, our defense substantially curtails the performance degradation induced by poisoned updates, thereby preserving the robustness of the global model against large-scale adversarial influence.

**Figure 16:**
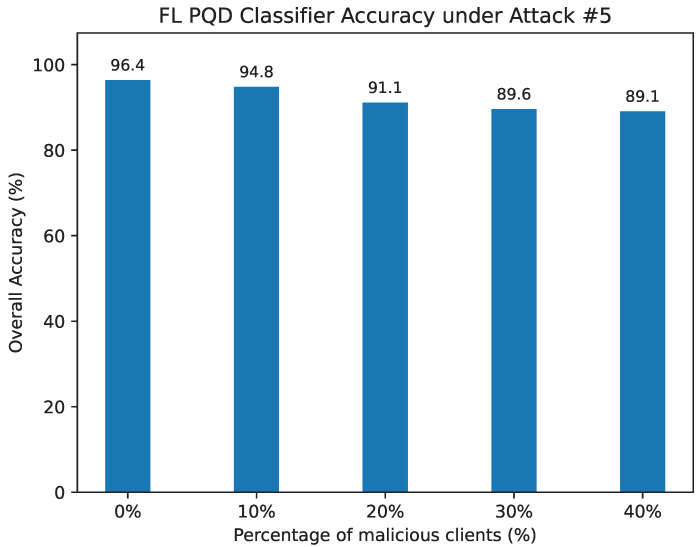
The FL PQD classifier accuracy under Attack #5 using the Median Defense.

Attack #5. Table 10 and Figure 16 present the performance of the FL PQD classifier under Attack #5 when protected by our defense. The HD metric remains robust, showing only a modest reduction from 92.5% in the benign case to 84.19% with 40% malicious clients. In contrast, without defense (Table 6), HD collapses immediately, reaching 0% as soon as adversarial updates are introduced.

A similar stabilizing effect is observed across precision, F1-score, and false alarm rate, where the defense curtails the sharp degradation evident in the undefended setting. Overall classification accuracy is likewise preserved, decreasing from 96.4% with no attackers to 89.09% at 40% adversarial participation. By comparison, the unprotected model suffers a dramatic breakdown, as shown in Figure 10, where accuracy plunges from 96% to only 5.88% with the involvement of a single malicious client.

These results highlight the resilience of our defense, demonstrating its effectiveness in sustaining classifier reliability even under highly adversarial conditions.

## 7. Conclusions

Machine learning has proven to be an effective method for identifying PQDs in smart grids—an essential step for enabling timely responses and preventing grid malfunctions or degraded performance. In this study, we began by training three PQD classifiers using different architectures: a CNN, a RNN with LSTM units, and a hybrid CNN-RNN model. This initial phase was conducted using a real-world dataset under a centralized learning setup, assuming all data is available to a single party. The goals were twofold: (1) to determine the best-performing architecture for subsequent experiments, and (2) to establish a performance baseline.

Following this, we moved to a FL scenario, focusing on the hybrid CNN-RNN model. Unlike centralized training, FL enables collaborative learning without sharing raw data, which is advantageous in cases where access to large, diverse datasets is practically constrained. As expected from prior FL research, we observed a slight drop in performance: the model’s accuracy decreased from 97% (centralized) to 96% (FL), while the false alarm rate increased from 0.19% to 4%. In this phase, we assumed all participating clients were honest. These results serve as a reference for later experiments involving adversarial clients.

Next, we evaluated the impact of malicious participants by simulating five types of adversarial attacks. The first attack was targeted, designed to disrupt the model’s ability to classify a specific disturbance. The remaining four were untargeted, aiming to generally reduce classification accuracy. In the targeted attack, we applied a model replacement strategy. This successfully reduced the classification accuracy for class #8—the target class—from 96% to nearly 0%, while leaving the accuracy of other classes largely unaffected.

Among the untargeted attacks, Attack #2, which involved random relabeling of data by malicious clients, had limited success. With 40% of clients acting maliciously, accuracy dropped from 96% to 89.9%. Attack #3 improved upon this by using clustering to select data samples less likely to overlap with those of honest clients, thereby reducing their influence. This approach was more effective at higher proportions of malicious participants. Attack #4, in contrast to the previous ones, altered input features instead of labels and demonstrated greater success, though its impact was still modest when the attacker ratio was low.

Finally, Attack #5, which used the model replacement technique, proved the most effective among untargeted attacks—especially in scenarios with only a few adversaries. Unlike the other attacks, this strategy allows even a single malicious client to override the global model update, making it significantly more potent when attackers are few.

To counter these attacks, we apply a defense that detects and removes poisoned updates before aggregation, preventing them from corrupting the global model. The method relies on the fact that malicious updates deviate markedly from the typical patterns of honest clients. By measuring pairwise distances between submitted models, the algorithm flags and filters out likely outliers. Experiments show that this approach effectively restores the global model’s performance to near attack-free levels.

While this paper focuses on poisoning attacks, our future work will extend the investigation to include other adversarial threats, such as Trojan (backdoor) attacks, and to explore the hierarchical federated learning (HFL) architecture. Trojan attacks occur during training when an attacker implants a hidden rule in the model so that inputs containing a specific, subtle trigger cause incorrect classification while maintaining normal behavior on clean data. These attacks are particularly dangerous in federated settings because malicious clients can inject poisoned updates without revealing raw data, and non-IID distributions or secure aggregation can conceal anomalies. Defending against Trojans requires layered strategies, including robust aggregation, anomaly detection, input sanitization, and post-deployment unlearning. In parallel, HFL extends standard federated learning by organizing clients and servers into multiple layers—such as edge, regional, and global aggregators—allowing local updates to be aggregated hierarchically before reaching the central server. This structure improves scalability, reduces communication overhead, supports privacy within organizational boundaries, and enhances robustness in large, distributed systems such as smart grids, IoT networks, and multi-institution collaborations.

## Figures and Tables

**Table 1:** Descriptions of Power Quality Disturbance Classes.

Disturbance Type	Description
Pure_Sinusoidal	Ideal undistorted sinusoidal waveform with constant amplitude and frequency.
Sag	Short-duration reduction in voltage magnitude below the nominal value.
Swell	Short-duration increase in voltage magnitude above the nominal value.
Interruption	Complete loss of voltage for a short period.
Transient	Sudden, non-periodic voltage spike or dip.
Oscillatory_Transient	Short-duration oscillations superimposed on the waveform, often decaying over time.
Harmonics	Presence of sinusoidal components at integer multiples of the fundamental frequency.
Harmonics_with_Sag	Combination of harmonic distortion and a voltage sag event.
Harmonics_with_Swell	Combination of harmonic distortion and a voltage swell event.
Flicker	Low-frequency modulation of voltage amplitude, causing visible light fluctuations.
Flicker_with_Sag	Voltage flicker occurring simultaneously with a sag event.
Flicker_with_Swell	Voltage flicker occurring simultaneously with a swell event.
Sag_with_Oscillatory_Transient	Voltage sag accompanied by oscillatory transient components.
Swell_with_Oscillatory_Transient	Voltage swell accompanied by oscillatory transient components.
Sag_with_Harmonics	Voltage sag occurring with harmonic distortion.
Swell_with_Harmonics	Voltage swell occurring with harmonic distortion.
Notch	A sharp, brief drop in voltage typically caused by commutation in power electronic devices.

**Table 2:** Performance comparison of different deep learning models.

	Total Parameters	ACC	PR	F1	Recall	FA	HD
CNN	107,791	92.86%	92.81%	92.72%	92.73%	0.45%	92.28%
RNN (LSTM)	61,201	68.5%	69.93%	67.86%	68.57%	1.97%	66.6%
CNN + RNN	145,073	97%	96.95%	96.84%	96.83%	0.19%	96.64%

**Table 3:** Performance metrics of FL PQD classifier accuracy with different percentages of malicious clients for Attack #2.

Percentage of Malicious	No Attack	10% Malicious	20% Malicious	30% Malicious	40% Malicious
HD	92%	90.6%	87.6%	86.4%	80.6%
PR	95%	95%	94.1%	93.5%	90.7%
F1	94%	95%	93.4%	92.9%	89.8%
FA	4%	4.5%	5.9%	6.3%	9.3%

**Table 4:** Performance metrics of FL PQD classifier accuracy with different percentages of malicious clients for Attack #3.

Percentage of Malicious	No Attack	10% Malicious	20% Malicious	30% Malicious	40% Malicious
HD	92%	87.3%	80%	67.2%	21.5%
PR	95%	93%	90%	85%	54%
F1	94%	93%	89%	84%	43%
FA	4%	5%	9%	15%	21%

**Table 5:** Performance metrics of FL PQD classifier accuracy with different percentages of malicious clients for Attack #4.

Percentage of Malicious	No Attack	10% Malicious	20% Malicious	30% Malicious	40% Malicious
HD	92%	72.5%	52.5%	25.2%	2.4%
PR	95%	84%	78%	62%	49%
F1	94%	83%	70%	57%	41%
FA	4%	16%	22%	32%	45%

**Table 6:** Performance metrics of FL PQD classifier accuracy with different percentages of malicious clients for Attack #5.

Percentage of Malicious	No Attack	10% Malicious	20% Malicious	30% Malicious	40% Malicious
HD	92%	0%	0%	0%	0%
PR	95%	0%	0%	0%	0%
F1	94%	2%	1%	1%	0%
FA	4%	6%	6%	6%	6%

**Table 7:** Performance metrics of FL PQD classifier accuracy with different percentages of malicious clients for Attack #2 using the Median defense.

Percentage of Malicious	No Attack	10% Malicious	20% Malicious	30% Malicious	40% Malicious
HD	92.26%	91.95 %	89.96%	90.8%	89.08%
PR	95.57%	95.83%	94.06%	94.96%	93.69%
F1	95.3%	95.7%	94.09%	94.83%	93.38%
FA	4%	4.02%	4.1%	4.05%	4.3%

**Table 8:** Performance metrics of FL PQD classifier accuracy with different percentages of malicious clients for Attack #3 using the Median defense.

Percentage of Malicious	No Attack	10% Malicious	20% Malicious	30% Malicious	40% Malicious
HD	92.46%	91 %	88.44%	86.49%	86.05%
PR	96.42%	95.18%	93.36%	92.5%	91.42%
F1	96.32%	95.03%	92.93%	90.8%	90.7%
FA	3.9%	4%	4.5%	4.63%	4.8%

**Table 9:** Performance metrics of FL PQD classifier accuracy with different percentages of malicious clients for Attack #4 using the Median defense.

Percentage of Malicious	No Attack	10% Malicious	20% Malicious	30% Malicious	40% Malicious
HD	92.49%	92 %	91%	90.35%	88%
PR	96.6%	96.13%	95.52 %	95.01%	93.3%
F1	96.7%	96%	95.4%	94.9%	92.8%
FA	4.01%	4%	4.2%	4.45%	4.6%

**Table 10:** Performance metrics of FL PQD classifier accuracy with different percentages of malicious clients for Attack #5 using the Median defense.

Percentage of Malicious	No Attack	10% Malicious	20% Malicious	30% Malicious	40% Malicious
HD	92.5%	90.7 %	86.53%	84.83%	84.19%
PR	96.5%	95.06%	92.4 %	90.4%	89.61%
F1	96.3%	94.9%	90.98%	89.48%	88.97%
FA	3.9%	4.1%	4.56%	4.78%	4.9%

## Data Availability

All links to the data used in the study are cited in the text.
